# Immediate Risk for Cardiovascular Events and Suicide Following a Prostate Cancer Diagnosis: Prospective Cohort Study

**DOI:** 10.1371/journal.pmed.1000197

**Published:** 2009-12-15

**Authors:** Katja Fall, Fang Fang, Lorelei A. Mucci, Weimin Ye, Ove Andrén, Jan-Erik Johansson, Swen-Olof Andersson, Pär Sparén, Georg Klein, Meir Stampfer, Hans-Olov Adami, Unnur Valdimarsdóttir

**Affiliations:** 1 Department of Medical Epidemiology and Biostatistics, Karolinska Institutet, Stockholm, Sweden; 2 Department of Epidemiology, Harvard School of Public Health, Boston, Massachusetts, United States of America; 3 Channing Laboratory, Department of Medicine, Harvard Medical School, Boston, Massachusetts, United States of America; 4 Örebro University Hospital, Örebro, Sweden; 5 The Microbiology Tumor Biology Center, Karolinska Institutet, Stockholm, Sweden; 6 Centre of Public Health Sciences, University of Iceland, Reykjavík, Iceland; McGill University, Canada

## Abstract

**Background:**

Stressful life events have been shown to be associated with altered risk of various health consequences. The aim of the present study was to investigate whether the emotional stress evoked by a prostate cancer diagnosis increases the immediate risks of cardiovascular events and suicide.

**Methods and Findings:**

We conducted a prospective cohort study by following all men in Sweden who were 30 y or older (*n* = 4,305,358) for a diagnosis of prostate cancer (*n* = 168,584) and their subsequent occurrence of cardiovascular events and suicide between January 1, 1961 and December 31, 2004. We used Poisson regression models to calculate relative risks (RRs) and 95% confidence intervals (CIs) of cardiovascular events and suicide among men who had prostate cancer diagnosed within 1 y to men without any cancer diagnosis. The risks of cardiovascular events and suicide were elevated during the first year after prostate cancer diagnosis, particularly during the first week. Before 1987, the RR of fatal cardiovascular events was 11.2 (95% CI 10.4–12.1) during the first week and 1.9 (95% CI 1.9–2.0) during the first year after diagnosis. From 1987, the RR for cardiovascular events, nonfatal and fatal combined, was 2.8 (95% CI 2.5–3.2) during the first week and 1.3 (95% CI 1.3–1.3) during the first year after diagnosis. While the RR of cardiovascular events declined, the RR of suicide was stable over the entire study period: 8.4 (95% CI 1.9–22.7) during the first week and 2.6 (95% CI 2.1–3.0) during the first year after diagnosis. Men 54 y or younger at cancer diagnosis demonstrated the highest RRs of both cardiovascular events and suicide. A limitation of the present study is the lack of tumor stage data, which precluded possibilities of investigating the potential impact of the disease severity on the relationship between a recent diagnosis of prostate cancer and the risks of cardiovascular events and suicide. In addition, we cannot exclude residual confounding as a possible explanation.

**Conclusions:**

Men newly diagnosed with prostate cancer are at increased risks for cardiovascular events and suicide. Future studies with detailed disease characteristic data are warranted.

*Please see later in the article for the Editors' Summary*

## Abstract 2

Katja Fall and Fang Fang and colleagues find that men newly diagnosed with prostate cancer are at increased risk of cardiovascular events and suicide.

## Editors' Summary

**Background:**

Prostate cancer—a type of tumor that develops in a walnut-sized structure in the male reproductive system—is the commonest cancer (excluding skin cancer) among men in developed countries. In the USA and the UK, for example, one in six men will develop prostate cancer during their lifetime. Most prostate cancers develop in elderly men and, because these tumors usually grow relatively slowly, many men die with prostate cancer rather than as a result of it. Nevertheless, some prostate cancers are fast-growing and aggressive and prostate cancer is the second leading cause of cancer-related death among men. The symptoms of prostate cancer include problems urinating and excessive urination during the night. Nowadays, however, most prostate cancers are detected before they produce any symptoms by measuring the amount of a protein called the prostate-specific antigen (PSA) in the blood.

**Why Was This Study Done?:**

Widespread PSA screening was introduced 20 years ago in the hope that early detection of prostate cancer would save lives. But, although many more prostate cancers are detected nowadays, the number of prostate cancer deaths has not changed significantly. Experts are divided, therefore, about whether the potential benefits of PSA screening outweigh its risks. Treatments for prostate cancer (for example, surgical removal of the prostate) may be more effective if they are started early but they can cause impotence and urinary incontinence, so should men be treated whose cancer might otherwise never affect their health? In addition, receiving a diagnosis of prostate cancer is stressful and there is growing evidence that stressful life events can increase an individual's risk of becoming ill or dying from a heart attack, stroke, or other “cardiovascular” events and of becoming mentally ill. In this study, therefore, the researchers investigate whether men diagnosed with prostate cancer in Sweden have increased risks of cardiovascular events and suicide during the first week and first year after their diagnosis.

**What Did the Researchers Do and Find?:**

The researchers identified nearly 170, 000 men diagnosed with prostate cancer between 1961 and 2004 among Swedish men aged 30 years or older by searching the Swedish Cancer Register. They obtained information on subsequent fatal and nonfatal cardiovascular events and suicides from the Causes of Death Register and the Inpatient Register (in Sweden, everyone has a unique national registration number that facilitates searches of different health-related Registers). Before 1987, men with prostate cancer were about 11 times as likely to have a fatal cardiovascular event during the first week after their diagnosis as men without prostate cancer; during the first year after their diagnosis, men with prostate cancer were nearly twice as likely to have a cardiovascular event as men without prostate cancer (a relative risk of 1.9). From 1987, the relative risk of combined fatal and nonfatal cardiovascular events associated with a diagnosis of prostate cancer was 2.8 during the first week and 1.3 during the first year after diagnosis. The relative risk of suicide associated with a diagnosis of prostate cancer was 8.4 during the first week and 2.6 during the first year after diagnosis throughout the study period. Finally, men younger than 54 years at diagnosis had higher relative risks of both cardiovascular events and suicide.

**What Do These Findings Mean?:**

These findings suggest that men newly diagnosed with prostate cancer have an increased risk of cardiovascular events and suicide. Because there is no information on tumor size or aggressiveness in the Cancer Register, the researchers could not look at the relationship between disease severity and the likelihood of a cardiovascular event or suicide. Furthermore, because of the study design, men who received a diagnosis of prostate cancer may have had additional characteristics in common that contributed to their increased risk of cardiovascular events and suicide. Nevertheless, these findings strongly suggest that the stress of the diagnosis itself rather than any subsequent treatment has deleterious effects on the health of men receiving a diagnosis of prostate cancer. Thus, strategies should be developed to reduce the risks of cardiovascular events and suicide—increased clinical and psychological monitoring—after a diagnosis of prostate cancer, particularly among young men, and this new information should be considered in the ongoing debate about the risks and benefits of PSA screening.

**Additional Information:**

Please access these Web sites via the online version of this summary at http://dx.doi.org/10.1371/journal.pmed.1000197.

- The US National Cancer Institute provides information on all aspects of prostate cancer, (in English and Spanish)
- The US Centers for Disease Control and Prevention provides information on prostate cancer, including Prostate Cancer Screening, A Decision Guide (some information in multiple languages)
- The UK National Health Service Choices Web site provides detailed information on prostate cancer
- The UK-based Samaritans charity provides confidential nonjudgmental emotional support, 24 hours a day, for people who are experiencing feelings of distress or despair, including those which could lead to suicide
- Outside the UK, Befrienders provides information on help lines for those experiencing distress

## Introduction

Growing evidence suggests that stressful events, such as the loss of a child, war, or natural disasters, lead to an increased risk of psychiatric hospitalizations [1] and cardiovascular morbidity [2] with excess mortality [3]–[6]. Less is known about whether emotional stress evoked by a cancer diagnosis increases the risks of cardiovascular events and suicide, especially immediately after diagnosis. Such possible consequences are of specific interest for prostate cancer, which is now the most common malignancy among men in westernized countries. Widespread prostate-specific antigen (PSA)-testing has further entailed detection of large numbers of men with uncertain survival benefit [7],[8]. The increased diagnostic activity calls for new knowledge on possible stress-induced health effects that may be caused by the diagnosis. Using the nationwide Swedish population-based registers, we assessed whether men diagnosed with prostate cancer were at increased risks of cardiovascular events and suicide during the year after their diagnosis, in particular during the first weeks following diagnosis.

## Methods

We utilized the Swedish Census data that cover virtually all residents in Sweden in 1960, 1970, 1980, and 1990. We identified 4,305,358 men, born in Sweden and at age 30 y or older between January 1, 1961 and December 31, 2004. Using the national registration number, an individually unique identifier of all Swedish inhabitants, we were able to link Census information with the Swedish Cancer, Causes of Death, and Inpatient Registers and calculate incidence rates of cardiovascular events and suicide. The study was approved by the Regional Ethics Committee at the Karolinska Institutet.

### Diagnosis of Prostate Cancer

By linkage to the nationwide Swedish Cancer Register, we identified 173,701 men diagnosed with prostate cancer during the study period. Reporting of cancers by clinicians and pathologists is required by law since cancer registration began in 1958, and the completeness of the register approaches 100% [9]. A total of 5,117 men diagnosed with prostate cancer first at autopsy were excluded, leaving 168,584 men in the main analyses.

### Assessment of Outcome

We obtained information on cardiovascular death and suicide from the Causes of Death Register during the entire study period. The essentially complete Causes of Death Register, established in 1952, includes the date of death and information on underlying and contributory causes of death. Beginning for the year 1987, we were also able to ascertain nonfatal cardiovascular events from linkage with the Inpatient Register. Described in detail previously [10], the Inpatient Register was initiated primarily for administrative purposes in 1964, and its national coverage increased from 60% in 1969, to 85% in 1983, and to 100% in 1987. The Register data include the national registration number, up to eight discharge diagnoses, and time of admission/discharge. Cardiovascular events were registered as the underlying cause of death in the Causes of Death Register or the main diagnosis in the Inpatient Register according to the *International Classification of Diseases*; the 7th revision (ICD-7) was used for coding diagnoses through 1968, the 8th revision (ICD-8) between 1969 and 1986, the 9th revision (ICD-9) between 1987 and 1996, and the 10th revision (ICD-10) after 1996.

Between 1961 and 1986, cardiovascular events were defined as deaths with cardiovascular disease as the underlying cause registered in the Causes of Death Register. With the completeness of the Inpatient Register in 1987, cardiovascular events were defined as hospitalizations with cardiovascular disease as the main diagnosis (Inpatient Register) or deaths with cardiovascular disease as the underlying cause of death (Causes of Death Register), whichever came first. The detailed classification and codes for cardiovascular events are shown in the Table S1. The completeness and accuracy of the Inpatient Register are generally high. The false negative rate of ischemic heart disease was estimated to be 7% and the false positive rate was estimated to be 2% [11]. Deaths by suicide were classified in ICD-7 with codes E971–E979 and E9639, in ICD-8 and 9 with codes E950–959, and in ICD-10 with codes X60–X84 and Y870. Although some underreporting of suicide may be expected, a validation study reviewing forensic data indicates generally high accuracy of injury-related registration of causes of death [12].

To examine the potential heterogeneity between fatal and nonfatal cardiovascular events, we separately calculated the relative risks (RRs) of hospitalization for cardiovascular diseases and death from cardiovascular events among the prostate cancer patients diagnosed in 1987–2004. We further utilized the Inpatient Register to conduct stratified analyses based on previous hospitalizations for psychiatric (ICD-9 codes 290–316; ICD-10 codes F00–F99) and cardiovascular disorders among men later diagnosed with prostate cancer between 1990 and 2004. To address the possibility that cardiovascular outcomes might be due to the invasive procedures leading to the diagnosis of prostate cancer, we also performed several sensitivity analyses by excluding patients that were diagnosed at transurethral resection of the prostate, with any hospitalization during the month before diagnosis, or treated with radical prostatectomy after diagnosis, respectively.

### Follow-up

Follow-up of the study participants started from January 1, 1961 or from their 30th birthday, whichever came earlier. For those who did not develop prostate cancer, the follow-up ended at the date of diagnosis of any other malignancy, death, emigration, or December 31, 2004, whichever occurred first; accumulated person-time was defined as unexposed person-time. For men diagnosed with prostate cancer, the follow-up ended 1 y after the diagnosis, occurrence of cardiovascular events or suicide, other death, emigration out of Sweden, or December 31, 2004, whichever came first; accumulated person-time defined as exposed person-time. Men diagnosed with prostate cancer contributed unexposed person-time before the date of diagnosis (as recorded in the Cancer Register) and exposed person-time thereafter.

To examine the RRs of suicide and cardiovascular events beyond the first year after prostate cancer diagnosis, we performed additional analysis by extending the follow-up among prostate cancer patients beyond the first year time window until the occurrence of cardiovascular events or suicide, other death, emigration, or end of study.

### Statistical Analyses

We used log-linear Poisson regression models to calculate the overall RRs and 95% confidence intervals (95% CIs) of cardiovascular events and suicide, as the ratio of the corresponding incidence rates among the newly diagnosed prostate cancer patients to the incidence rates among the cancer-free men. We adjusted for calendar period and age in all statistical models. We compared the RRs within strata of age, calendar year of follow-up, and time since diagnosis (week 1, weeks 2–4, weeks 5–26, and weeks 27–52). During the period from 1990 through 2004, we calculated RR for suicide by marital status, educational level (as registered in the Swedish Education Register, which contains educational information of Swedish residents alive in 1985 and onward), and history of psychiatric hospitalization. We also compared RRs of cardiovascular events among prostate cancer patients diagnosed from 1990 through 2004 after stratification by history of cardiovascular hospitalization. The significance of potential interactions between the above variables and prostate cancer diagnosis was tested by adding a term of one specific variable multiplying prostate cancer diagnosis. Pearson's χ^2^ test was used to check the goodness of fit of the models. Overdispersion was corrected by using the deviance factor of Pearson's statistic if any.

The overall RRs of cardiovascular events and suicide were also calculated for follow-up beyond 1 y after diagnosis among the newly diagnosed prostate cancer patients. For comparison, we also calculated the RR of hospitalization for two other common conditions in this population (osteoarthritis [ICD-9: 715A–D, 715W, 715X; ICD-10: M15–19] and diabetes [ICD-9: 250; ICD-10: E10–14]) following the diagnosis of prostate cancer. For the statistical analyses we used SAS version 9.1 software (SAS Institute, Inc.).

## Results

Between 1961 and 2004, a total of 4,305,358 men were followed with over 90 million unexposed person-years and approximately 150,000 person-years among men newly diagnosed with prostate cancer. The mean age at diagnosis of prostate cancer was 73.4 y (range 31.6–102.5 y). Of 168,584 men diagnosed with prostate cancer, 10,126 (6%) experienced a cardiovascular event during the year following diagnosis and 136 (0.08%) committed suicide.

### Cardiovascular Events

In 1961–1986, 4,631 of the 71,818 prostate cancer patients died from a cardiovascular event within 1 y after diagnosis; the risk for a fatal cardiovascular event among the prostate cancer patients was 1.9 times higher than among the cancer-free men (Table 1). The RR was highest during the first week after diagnosis (RR 11.2), decreased gradually thereafter, but remained elevated throughout the first year. The excess risk was greatest among men 54 y or younger at prostate cancer diagnosis (RR 4.8) but still elevated among men 75 y or older (RR 1.7). The RR tended to decrease with calendar time, from RR of 2.9 in 1961–1970 to RR of 1.3 in 1981–1986. The RR of fatal cardiovascular events was 1.1 (95% CI 1.1–1.1) beyond the first year of follow-up in 1961–1986.

**Table 1:** Incidence rates and RRs of cardiovascular events during the first year after the diagnosis of prostate cancer in Sweden, 1961–1986.

Category		Cardiovascular Events	IR per 1,000 Person-years	RR^a^ (95% CI)
**Totals**	**Cancer-free**	564,740	10.4	1.0
	**PCa**	4,631	77.1	1.9 (1.9–2.0)
**Time since diagnosis (wk)**				
**1**		710	457.9	11.2 (10.4–12.1)
**2–4**		848	215.6	5.4 (5.0–5.8)
**5–26**		1,729	65.0	1.6 (1.6–1.7)
**27–52**		1,344	48.0	1.2 (1.1–1.3)
**Age (y)**				
**≤54**	**Cancer-free**	57,423	1.6	1.0
	**PCa**	64	20.2	4.8 (3.7–6.0)
**55–64**	**Cancer-free**	121,544	12.0	1.0
	**PCa**	555	35.2	2.7 (2.5–2.9)
**65–74**	**Cancer-free**	200,113	33.7	1.0
	**PCa**	2,050	72.6	2.1 (2.0–2.2)
**≥75**	**Cancer-free**	185,660	94.7	1.0
	**PCa**	1,962	151.9	1.7 (1.6–1.8)
**Calendar year of follow-up**				
**1961–1970**	**Cancer-free**	199,397	9.8	1.0
	**PCa**	1,822	116.1	2.9 (2.8–3.0)
**1971–1980**	**Cancer-free**	228,847	10.9	1.0
	**PCa**	1,810	71.7	1.8 (1.7–1.9)
**1981–1986**	**Cancer-free**	136,496	10.5	1.0
	**PCa**	999	52.2	1.3 (1.2–1.4)

a Adjusted for age at follow-up (5-y group between 30 and 84, and ≥85 y) and calendar year (1-y group between 1961 and 1986).

IR, incidence rate; PCa, prostate cancer.

In 1987–2004, a total of 5,495 cardiovascular events were observed among the prostate cancer patients during the first year after diagnosis. The overall risk of fatal and nonfatal cardiovascular events was elevated among the prostate cancer patients (RR 1.3), especially during the first week after diagnosis (RR 2.8) (Table 2). As noted in 1961–1986, although the RR was strongest during the first week, it remained statistically significantly elevated throughout the first year after diagnosis. The excess risk was again greatest among men 54 y or younger at cancer diagnosis (RR 1.5). The magnitude of the RR declined over the calendar time and reached a plateau at around 20%–30% since the 1980s. Beyond the first year after diagnosis, the RR of overall cardiovascular events was 1.2 (95% CI 1.2–1.2) in 1987–2004.

**Table 2:** Incidence rates and RRs of cardiovascular events during the first year after the diagnosis of prostate cancer in Sweden, 1987–2004.

Category		Cardiovascular Events	IR per 1,000 Person-years	RR^a^ (95% CI)
**Totals**	**Cancer-free**	763,321	21.3	1.0
	**PCa**	5,495	82.6	1.3 (1.3–1.3)
**Time since diagnosis (wk)**				
**1**		279	176.5	2.8 (2.5–3.2)
**2–4**		429	104.2	1.7 (1.5–1.8)
**5–26**		2,321	79.8	1.3 (1.2–1.3)
**27–52**		2,466	77.7	1.2 (1.2–1.2)
**Age (y)**				
**≤54**	**Cancer-free**	184,128	7.1	1.0
	**PCa**	128	23.2	1.5 (1.3–1.8)
**55–64**	**Cancer-free**	177,070	33.9	1.0
	**PCa**	961	50.7	1.5 (1.4–1.6)
**65–74**	**Cancer-free**	232,084	66.7	1.0
	**PCa**	2,433	86.2	1.3 (1.2–1.4)
**≥75**	**Cancer-free**	170,039	122.0	1.0
	**PCa**	1,973	142.5	1.2 (1.1–1.2)
**Calendar year of follow-up**				
**1987–1992**	**Cancer-free**	338,670	27.4	1.0
	**PCa**	2,195	111.2	1.3 (1.3–1.4)
**1993–1998**	**Cancer-free**	236,947	20.1	1.0
	**PCa**	1,748	87.9	1.3 (1.3–1.4)
**1999–2004**	**Cancer-free**	187,704	16.0	1.0
	**PCa**	1,552	57.7	1.2 (1.2–1.3)

a Adjusted for age at follow-up (5-y group between 30 and 84, and ≥85 y) and calendar year (1-y group between 1987 and 2004).

IR, incidence rate; PCa, prostate cancer.

Among patients diagnosed in 1987–2004, the RR of hospitalization for cardiovascular diseases was 1.3 (95% CI 1.3–1.4) during the first year and 2.4 (95% CI 2.1–2.7) during the first week after diagnosis; the corresponding RRs for cardiovascular death were 1.1 (95% CI 1.1–1.1) during the first year and 3.7 (95% CI 3.2–4.2) during the first week.

Our data further suggest that men without a history of cardiovascular disease before the diagnosis of prostate cancer may have a higher RR of cardiovascular events immediately after a prostate cancer diagnosis compared to men with a history of cardiovascular disease (Table 3). Among men without a history of cardiovascular disease, prostate cancer diagnosis induced a 5-fold risk of cardiovascular event (fatal and nonfatal combined) in the first week (RR 4.8) and a 3-fold risk in the first 4 wk (RR 2.9) after prostate cancer diagnosis. Among men with previous cardiovascular history, the first-week RR was 2.8 and the first-4-wk RR was 1.8. After overdispersion correction, a borderline significant interaction between a history of cardiovascular disease and prostate cancer diagnosis was noted for the first-4-wk RR of overall cardiovascular events (*p* = 0.08) although not for the first week (*p* = 0.22).

**Table 3:** RRs of death from specific cardiovascular events during the first week and the first 4 wk after the diagnosis of prostate cancer by history of cardiovascular disease in Sweden, 1990–2004.

Category	All Cardiovascular Events		Myocardial Infarction	Embolism/Thrombosis	Other Heart Disease	Acute Cerebro-Vascular Events
	*n*	RR (95% CI)	RR (95% CI)	RR (95% CI)	RR (95% CI)	RR (95% CI)
**Men without a history of cardiovascular events**						
**Cancer-free**	51,378	1.0	1.0	1.0	1.0	1.0
**PCa, 1 wk after diagnosis**	38	4.8 (3.2–6.9)	4.8 (2.8–7.5)	18.3 (7.1–37.5)	5.9 (1.9–14.9)	—
**PCa, 4 wk after diagnosis**	84	2.9 (2.2–3.7)	2.9 (2.0–3.9)	10.1 (4.3–19.9)	2.7 (1.2–5.7)	3.5 (1.3–7.1)
**Men with a history of cardiovascular events**						
**Cancer-free**	204,627	1.0	1.0	1.0	1.0	1.0
**PCa, 1 wk after diagnosis**	116	2.8 (2.0–3.8)	3.9 (2.9–5.1)	7.9 (3.6–14.7)	4.8 (2.4–8.3)	1.3 (0.4–3.3)
**PCa, 4 wk after diagnosis**	265	1.8 (1.4–2.2)	2.1 (1.4–2.2)	4.0 (2.0–7.0)	2.4 (1.4–3.8)	1.1 (0.6–1.9)

Analysis restricted to patients diagnosed since January 1, 1990 (3 y after the nationwide completion of the Inpatient Register) and adjusted for age at follow-up (5-y group between 30 and 84, and ≥85 y) as well as calendar year (5-y group between 1990–2004).

PCa, prostate cancer.

To assess whether the higher risk for cardiovascular events after prostate cancer diagnosis was due to better cancer detection among men with previous heart disease or deteriorating health in general, we excluded all men with any hospitalization during the month before prostate cancer diagnosis. This restriction did not alter the results (unpublished data). Similarly, exclusion of men with radical prostatectomy or transurethral resection of the prostate did not affect the RR estimates (unpublished data). Finally, men diagnosed with prostate cancer did not show an increased risk of being hospitalized for osteoarthritis (RR 1.0; 95% CI 0.8–1.4) or diabetes (RR 0.8; 95% CI 0.5–1.5) during the first month after their diagnosis.

### Suicide

A total of 136 (0.08%) prostate cancer patients committed suicide within 1 y after cancer diagnosis; the risk for suicide was more than doubled among prostate cancer patients compared to the cancer-free men (RR 2.6) (Table 4). The excess risk was highest during the first week after diagnosis (RR 8.4) and decreased with time since diagnosis, but was still clearly significant by the end of the first year (RR 1.9). The RR for suicide after diagnosis was 4.6 among men 54 y or younger, approximately twice that of older age groups. The risk of suicide remained more than doubled throughout the entire study period and was not modified by educational level (*p* for interaction = 0.88), marital status (*p* for interaction = 0.20), or prior psychiatric hospitalization (*p* for interaction = 0.26) (unpublished data). The risk of suicide remained elevated beyond the first year of follow-up (RR 1.8; 95% CI 1.6–2.0) during the entire study period.

**Table 4:** Incidence rates and RRs of suicide during the first year after the diagnosis of prostate cancer in Sweden, 1961–2004.

Category		Suicide	IR per 1,000 person-years	RR^a^ (95% CI)
**Totals**	**Cancer-free**	31,822	0.3	1.0
	**PCa**	136	0.9	2.6 (2.1–3.0)
**Time since diagnosis (wk)**				
**1**		11	3.0	8.4 (1.9–22.7)
**2–4**		9	1.0	2.7 (1.3–4.8)
**5–26**		69	1.0	3.0 (2.3–3.7)
**27–52**		47	0.7	1.9 (1.4–2.4)
**Age at follow-up (y)**				
**≤54**	**Cancer-free**	20,687	0.3	1.0
	**PCa**	14	1.5	4.6 (2.6–7.4)
**55–64**	**Cancer-free**	5,524	0.3	1.0
	**PCa**	23	0.6	1.9 (1.2–2.8)
**65–74**	**Cancer-free**	3,862	0.4	1.0
	**PCa**	62	0.9	2.6 (2.0–3.4)
**≥75**	**Cancer-free**	1,749	0.4	1.0
	**PCa**	37	1.1	2.5 (1.8–3.4)
**Calendar year of follow-up**				
**1961–1970**	**Cancer-free**	7,818	0.4	1.0
	**PCa**	24	1.5	3.4 (2.2–5.0)
**1971–1980**	**Cancer-free**	8,174	0.4	1.0
	**PCa**	24	1.0	2.3 (1.6–3.1)
**1981–1990**	**Cancer-free**	7,452	0.3	1.0
	**PCa**	37	1.1	2.8 (1.7–4.2)
**1991–2004**	**Cancer-free**	8,378	0.3	1.0
	**PCa**	51	0.7	2.3 (1.8–2.8)

a Adjusted for age at follow-up (5-y group between 30 and 84, and ≥85 y) and calendar year (5-y group between 1961–2004).

IR, incidence rate; PCa, prostate cancer.

## Discussion

In this large population-based study, men newly diagnosed with prostate cancer were at higher risks of cardiovascular events and suicide. The excess risks were highest during the first week after diagnosis, suggesting that the stress of diagnosis itself rather than subsequent factors such as hormonal treatment or operations plays a role. Also, we observed the highest excess risk of cardiovascular events among younger men, for whom the potential loss of years to live, of sexual potency, and of other aspects of life might be of greater concern than in older men.

We propose that the emotional stress associated with the diagnosis of prostate cancer leads to higher risks of cardiovascular morbidity and suicide. To our knowledge, no study has previously explored whether cardiovascular morbidity increases immediately after the diagnosis of prostate cancer. Several lines of evidence support the notion that emotionally stressful life events may lead to stunning or altered function of the heart. Wittstein and colleagues described stress-related left ventricular dysfunction in 13 individuals who had experienced a stressful event [13]; the authors found an exaggerated sympathetic stimulation to be responsible for this reversible condition. Epidemiological studies have found further manifestations of cardiovascular morbidity due to emotional stress. Increased risk of myocardial infarction was documented following the Athens earthquake in 1983 [3]. Bereaved parents have been reported to have increased risk for both fatal and nonfatal myocardial infarction [2], and more recently emotional stress brought on by viewing a World cup soccer match was reported to raise the risk for cardiovascular morbidity and mortality [14]. Our data suggest that being diagnosed with prostate cancer may also serve as a stressor of substantial weight, resulting in severe health outcomes.

Although a statistically significant excess risk of cardiovascular events during the year after prostate cancer diagnosis was observed over the entire study period in our data, the magnitude of the excess risk declined from 2.9 in the 1960s toward 1.3 in 1980s and stayed stable onwards. The underlying reason for the declination before 1980s is unclear. The decreasing incidence of advanced prostate cancer over calendar time as well as the increasing awareness of prostate cancer in the general population may reduce the intensity of emotional stress experienced by men diagnosed with prostate cancer. However, the fact that the RR of suicide remained relatively stable during the entire study period challenges this explanation. Furthermore, the RR of cardiovascular events dropped already in the early 1980s, which was before the advent of PSA screening and many men were still diagnosed with advanced disease, and stayed stable thereafter. A more probable explanation for the decrease in the immediate RR of cardiovascular events, especially the fatal events, after prostate cancer diagnosis may be the closer contact with medical surveillance or increased use of cardiovascular therapy; for example, beta blockers may have provided protection against cardiovascular events particularly in individuals exposed to emotionally stressful events. In line with this reasoning, we noted that prostate cancer patients with a history of cardiovascular disease had a lower RR of cardiovascular events, compared to patients without a history of cardiovascular disease.

Although the absolute risk was small, our findings indicate that prostate cancer patients are at considerably higher risk of suicide, particularly during the first week after diagnosis. The magnitude of the RR for suicide stayed relatively consistent during the entire study period. Earlier studies have shown that stressful life events including loss of a child increase the risks of both psychiatric hospitalizations [1] and unnatural deaths [5]. Studies have also reported an increased risk of suicide among patients with cancer of the breast [15], prostate [16], and other sites [17]–[19]; few of them indicate that the risk may be highest during the first months or years after diagnosis [18],[19]. Our observation that the excess risk was most prominent during the first week after prostate cancer diagnosis has, to our knowledge, not been reported previously. Possible mechanisms remain unknown but the emotional shock caused by the diagnosis, anxiety for the pending choice of treatment, together with emotional isolation, may explain the impulsive action. About 20% of the prostate cancer patients were reported as having no one to confide in [20].

Strengths of our study include the large size, population-based design, prospective data collection, and complete follow-up, with minimal potential for bias or differential misclassification of exposure and outcome. There are several limitations. The date of diagnosis assessed from the Cancer Register represents the date of the pathology diagnosis; thus, the patients may be informed days or even weeks later after the registered time of diagnosis. While this would not alter the number of events, it might influence the risk estimates and chiefly entail underestimation of the true excess risk during the first week and overestimation of the excess risk during subsequent weeks. Based on the register data, our study lacked information on clinical features including tumor stage at diagnosis. The level of stress experienced by the patient at diagnosis, and correspondingly the risk of immediate hazardous outcomes, may vary with stage (or chances of cure) [21], and we believe the associations with immediate adverse health effects could be stronger among patients with advanced stage disease. The lack of stage information does not affect, however, the validity of our findings. Exclusion of men that were hospitalized for any condition during the month before diagnosis did not alter our results—thus alleviating concerns of reverse causality.

Furthermore, analyses excluding patients undergoing transurethral resection of the prostate or radical prostatectomy did not attenuate our RR estimates. Although the true number of suicides is difficult to assess even after close scrutiny of all available information [12], we lack evidence that misclassification of death would differ between men with and without a prostate cancer diagnosis. Given the observational design of the study, influence of unknown or unmeasured confounders cannot be ruled out.

While the incidence of prostate cancer has doubled over the last 30 y in Sweden as well as in other western countries, the mortality by prostate cancer remained unchanged [22] and whether PSA screening does more good than harm is hotly debated [7],[8],[23]. Our finding of an association between prostate cancer diagnosis and the increased occurrence of sudden cardiovascular events and suicide adds new knowledge to be weighed into this challenging issue. Limited to prostate cancer patients, our findings cannot be readily generalized to other cancer types or to women. The declining excess risk of cardiovascular events after prostate cancer diagnosis over calendar time shows how multiple factors that vary across time and cultures, for example, medical advancements and degree of stress at a cancer diagnosis, modify the risk of hazardous outcomes after a prostate cancer diagnosis. Thus, alertness in clinical care and careful monitoring of the psychological health of newly diagnosed prostate cancer patients is needed as well as development of strategies to reduce the risk of adverse health outcomes after diagnosis.

In summary, our data suggest that men who receive a prostate cancer diagnosis are immediately at increased risks of cardiovascular events and suicide. The risks are highest during the first week after diagnosis and young men seem to be most vulnerable. These unrecognized consequences of a prostate cancer diagnosis deserve the attention of health professionals who take care of the increasing number of men that are diagnosed with this disease.

## Supporting Information

Table S1 Classification of cardiovascular disease.
(0.04 MB DOC)

## Abbreviations

CI: confidence interval
ICD: International Classification of Diseases
PSA: prostate-specific antigen
RR: relative risk
