# Supplementary material for: Immediate Risk for Cardiovascular Events and Suicide Following a Prostate Cancer Diagnosis: Prospective Cohort Study
Source: PLoS Med. 2009 Dec 15;6(12):e1000197. doi: 10.1371/journal.pmed.1000197 (PMC2784954; doi:10.1371/journal.pmed.1000197)
Supplement: Table S1 — Classification of cardiovascular disease. (0.04 MB DOC) [file pmed.1000197.s001.doc]

| **Table S1. Classification of Cardiovascular Disease** | | | | | |
| --- | --- | --- | --- | --- | --- |
| **Broad Category** | **Specific Category** | **ICD 7** | **ICD 8** | **ICD 9** | **ICD 10** |
| MI | Acute Myocardial Infarction | 420: 10, 17-18 | 410-411 | 410-411 | I21, I23, I24 |
| Other heart disease | Hypertensive heart disease | 442-447 | 400-404 | 401-405 | I10-13 |
|  | Aortic and other artery aneurysms | 451-452 | 441,442 | 441 | I71-72 |
| Thrombosis/Emboli | Artery Thrombosis/Embolus  Pulmonary Emboli | 454, 453, 465 | 444, 450 | 444, 415 | I74, I26 |
|  | Thrombophlebitis | 463,99, 466 | 451-452 | 451B-453 | I80.1 – I82 |
|  | Esophagus varicae, bleeding | 462,10 | 456 | 456A,B | I85.0 |
| Acute Cerebrovascular Disease | Arachnoidal bleeding | 330 | 430 | 430 | I60 |
|  | Intracerebral bleeding  (hemorrhagic stroke) | 331 | 431 | 431 | I61-62 |
|  | Thrombosis/infarction  (ischemic stroke) | 332 | 432-34 | 433-434 | I63-I64 |
